# Does co-infection with vector-borne pathogens play a role in clinical canine leishmaniosis?

**DOI:** 10.1186/s13071-018-2724-9

**Published:** 2018-03-20

**Authors:** Marta Baxarias, Alejandra Álvarez-Fernández, Pamela Martínez-Orellana, Sara Montserrat-Sangrà, Laura Ordeix, Alicia Rojas, Yaarit Nachum-Biala, Gad Baneth, Laia Solano-Gallego

**Affiliations:** 1 Departament de Medicina i Cirurgia Animals, Facultat de Veterinària, Universitat Autònoma de Barcelona, Bellaterra, Spain; 2 Hospital Clínic Veterinari, Facultat de Veterinària, Universitat Autònoma de Barcelona, Bellaterra, Spain; 3 Koret School of Veterinary Medicine, Hebrew University, Rehovot, Israel

**Keywords:** Canine leishmaniosis, Spain, *Leishmania infantum*, *Rickettsia conorii*, *Ehrlichia canis*, *Anaplasma phagocytophilum*, *Anaplasma platys*, *Hepatozoon canis*, *Bartonella henselae*, Co-infection

## Abstract

**Background:**

The severity of canine leishmaniosis (CanL) due to *Leishmania infantum* might be affected by other vector-borne organisms that mimic its clinical signs and clinicopathological abnormalities. The aim of this study was to determine co-infections with other vector-borne pathogens based on serological and molecular techniques in dogs with clinical leishmaniosis living in Spain and to associate them with clinical signs and clinicopathological abnormalities as well as disease severity.

**Methods:**

Sixty-one dogs with clinical leishmaniosis and 16 apparently healthy dogs were tested for *Rickettsia conorii*, *Ehrlichia canis*, *Anaplasma phagocytophilum* and *Bartonella henselae* antigens by the immunofluorescence antibody test (IFAT) and for *E. canis*, *Anaplasma* spp., *Hepatozoon* spp., *Babesia* spp. and filarioid DNA by polymerase chain reaction (PCR).

**Results:**

Among the dogs examined by IFAT, the seroprevalences were: 69% for *R. conorii*, 57% for *E. canis*, 44% for *A. phagocytophilum* and 37% for *B. henselae*; while the prevalences found by PCR were: 8% for *Ehrlichia*/*Anaplasma*, 3% for *Anaplasma platys* and 1% for *H. canis*. No other pathogen DNA was detected. Statistical association was found between dogs with clinical leishmaniosis and seroreactivity to *R. conorii* antigen (Fisher’s exact test: *P* = 0.025, OR = 4.1, 95% CI = 1–17) and *A. phagocytophilum* antigen (Fisher’s exact test: *P* = 0.002, OR = 14.3, 95% CI = 2–626) and being positive to more than one serological or molecular tests (co-infections) (Mann-Whitney test: *U* = 243, *Z* = -2.6, *n*_*1*_ = 14, *n*_*2*_ = 61, *P* = 0.01) when compared with healthy dogs. Interestingly, a statistical association was found between the presence of *R. conorii*, *E. canis*, *A. phagocytophilum* and *B. henselae* antibodies in sick dogs and some clinicopathological abnormalities such as albumin and albumin/globulin ratio decrease and increase in serum globulins. Furthermore, seroreactivity with *A. phagocytophilum* antigens was statistically associated with CanL clinical stages III and IV.

**Conclusions:**

This study demonstrates that dogs with clinical leishmaniosis from Catalonia (Spain) have a higher rate of co-infections with other vector-borne pathogens when compared with healthy controls. Furthermore, positivity to some vector-borne pathogens was associated with more marked clinicopathological abnormalities as well as disease severity with CanL.

## Background

Canine leishmaniosis (CanL) is a zoonotic protozoan disease caused by *Leishmania infantum* endemic in the Mediterranean basin. *Phlebotomus* spp. sand flies are the only vector adapted for the biological transmission of *L. infantum* in Europe [1]. Dogs (*Canis familiaris*) are considered the main peridomestic reservoir of this parasite in endemic areas [2]. Prevalence of canine *L. infantum* infection can be as high as 67% in selected populations [3], but the prevalence of clinical disease is usually lower than 10% [4]. The most useful diagnostic methods of CanL include quantitative serological techniques and PCR, although the direct observation of amastigote forms of *Leishmania* spp. is also helpful in the clinical setting [4–6].

The clinical manifestations of CanL can vary from absence of clinical signs and clinicopathological abnormalities to a severe fatal clinical illness. The most common clinical signs are skin lesions, generalized lymphadenomegaly, progressive weight loss, decreased appetite, lethargy, muscular atrophy, exercise intolerance, splenomegaly, polyuria and polydipsia, ocular lesions, epistaxis, lameness, vomiting and diarrhea [2, 4, 6].

In the Mediterranean basin, other canine vector-borne diseases are common. Some studies have documented *Ehrlichia canis* [7–12], *Anaplasma platys* [10, 12] and *Rickettsia conorii* [8, 9, 13, 14] infections in dogs. These intracellular Gram-negative bacteria are transmitted or likely transmitted by *Rhipicephalus sanguineus* (*sensu* *lato*) ticks [14–19]. It has been reported that the prevalence of these vector-borne infections is higher in communal shelter dogs and dogs that live outdoors [9, 12]. The clinical characteristics of rickettsial disease in dogs can be similar to those caused by *L. infantum*. *Anaplasma phagocytophilum* [8, 9, 11, 12] is another canine pathogen transmitted by *Ixodes ricinus* ticks that can infect dogs and humans causing acute febrile illness or transient subclinical infection [12, 20]. Other protozoan pathogens such as *Babesia vogeli* [10] and *Hepatozoon canis* [10] infect dogs in the Mediterranean basin and are also transmitted by *R. sanguineus* (*s.l*.) ticks [15, 17, 18].

*Bartonella* spp. are vector-borne bacteria that cause diseases in humans and animals globally, including Europe [7, 8]. Numerous species of *Bartonella* have been identified as pathogenic to humans while *Bartonella vinsonii berkhoffii* and *Bartonella henselae* are associated with clinical disease in dogs [21]. Dirofilariasis due to *Dirofilaria immitis* or *Dirofilaria repens* is another vector-borne disease transmitted by mosquitoes from the family Culicidae present in Europe [8, 12, 22, 23] that can affect both animals and humans [24], causing cardiopulmonary or subcutaneous disease manifestations, respectively [24].

It has been reported that infections with other vector-borne organisms can affect the severity of CanL or mimic its clinical signs and/or clinicopathological abnormalities [25–27]. Some studies have described co-infection of *L. infantum* with other vector-borne diseases in dogs with typical signs of leishmaniosis [7, 10, 28, 29]. Other authors have demonstrated co-infections with *L. infantum* and *E. canis*, *A. phagocytophilum* and *Bartonella* spp. in the Mediterranean area [11, 26, 30, 31]. Mekuzas et al. [30] found that clinical signs were more frequent in dogs with dual *L. infantum* and *E. canis* infection than dogs with a single infection. Roura et al. [7] found that simultaneous infection with two or more pathogens should be expected in dogs living in areas which are highly endemic for several vector-borne pathogens, mainly in dogs kept predominantly outdoors and not regularly treated with ectoparacitides.

The aim of this study was to determine co-infections with other vector-borne pathogens in dogs with clinical leishmaniosis living in Catalonia (Spain) and to associate with clinical signs and clinicopathological abnormalities as well as with disease severity. These dogs were compared with healthy control dogs living in the same geographical area.

## Methods

### Dogs

The dogs included in this study were from Catalonia (Spain), an area endemic for CanL and other vector-borne diseases. Sixty-one sick dogs were diagnosed with clinical leishmaniosis based on compatible clinicopathological findings and a medium or high antibody level in a quantitative ELISA for the detection of *L. infantum*-specific antibodies and cytology or histology in some cases. Physical examination; a complete blood count (CBC) with blood smear examination (System Siemens Advia 120; Siemens Healthcare GmbH, Erlanger, Germany); a biochemical profile including creatinine, urea, total proteins, alanine transaminase (ALT) and total cholesterol (Analyzer Olympus AU 400; Beckman Coulter Inc., Brea, CA, USA); urinalysis with urinary protein creatinine ratio; and serum electrophoresis were performed. Reference intervals for the hematological and biochemical parameters were employed as previously described [32]. The dogs were examined at different veterinary centers: 33 were from Fundació Hospital Clínic Veterinari (Bellaterra, Barcelona, Spain), 15 were from Hospital Ars Veterinaria (Barcelona, Barcelona, Spain), 7 from Hospital Mediterrani Veterinaris (Reus, Tarragona, Spain) and 6 from Consultori Montsant (Falset, Tarragona, Spain). The following clinical signs were recorded: fever, weight loss, skin lesions, ocular lesions, lymphadenomegaly, muscular atrophy, splenomegaly, vomiting and diarrhea, joint pain, polyuria and polydipsia, lameness, epistaxis and neurological disorders. Lymphadenomegaly was classified as mild, moderate or marked depending on the relative size of the enlarged lymph node. Furthermore, the dogs were classified according to the LeishVet clinical staging system [4]. *Leishmania* real-time PCR (qPCR) was performed on blood from all dogs [33].

Sixteen apparently healthy dogs from Barcelona province, based on clinical history, normal physical examination, seronegative and qPCR-negative for *Leishmania* were also studied for comparison with the sick dogs. Healthy dogs were from Barcelona province and were also examined at the same veterinary centres.

### Samples

Six millilitres of blood were collected from the dogs by jugular or metatarsal venipuncture for the laboratory tests described above. Blood was transferred into different tubes: ethylenediaminetetraacetic acid (EDTA) tubes for hematology and molecular testing, heparin for whole blood assay and plain serum tubes for biochemistry and serological testing. Once collected, samples were left at 4 °C overnight and then frozen at -80 °C until further use.

All serum and whole blood extractions were performed at the time of diagnosis between 2014 and 2016 and stored at -80 °C until use for this study.

### Quantitative ELISA for the detection of *L. infantum*-specific antibodies

The in-house ELISA was performed on sera of all dogs studied as previously described [33]. All samples with an optical density (OD) equal or higher to three were studied using a two-fold serial dilution ELISA as described elsewhere [33].

### Whole blood assay and sandwich ELISAs for the detection of canine IFN-γ

Whole blood assay and sandwich ELISAs for the detection of canine IFN-γ were performed on blood of all dogs studied as previously described [34].

### IFAT for *Rickettsia conorii*, *Ehrlichia canis*, *Anaplasma phagocytophilum* and *Bartonella henselae* antigens

Indirect immunofluorescence assays for the detection of specific IgG antibody against *R. conorii* (MegaFLUO® RICKETTSIA conorii; Diagnostik Megacor, Hörbranz, Austria), *E. canis* (MegaFLUO® EHRLICHIA canis; Diagnostik Megacor. Hörbranz, Austria), *A. phagocytophilum* (MegaFLUO® ANAPLASMA phagocytophilum; Diagnostik Megacor. Hörbranz, Austria) and *B. henselae* (MegaFLUO® BARTONELLA henselae; Diagnostik Megacor. Hörbranz, Austria) antigens were performed on sera. IFATs were performed for 75 of the 77 dogs included in this study: 61 dogs with clinical leishmaniosis and 14 apparently healthy dogs. The samples were diluted to 1:64 with PBS and 20 μl of every serum dilution was applied per well. The slides were incubated for 30 min at 37 °C. After that, a washing procedure was performed. The slides were washed twice with PBS for 5 min and once with distilled water. After the washing procedure described, 15 μl of FLUO FITC anti-dog IgG conjugate was added into each well. The slides were incubated for another 30 min at 37 °C in the dark to protect the photosensitive conjugate. The washing procedure described above was repeated. After the second washing procedure, some drops of mounting medium were added on the cover slips. The slides were evaluated using a fluorescence microscope (Leica DM6000 B; Leica Microsystems, Wetzlar, Germany) at 200× and 400× magnification and each well was compared to the fluorescence pattern seen in the positive and negative controls. All samples were examined by three different investigators to avoid errors of observation. All samples negative at 1:64 were considered negative and no further dilutions were done.

All samples with a positive result were further investigated using a two-fold serial dilution IFAT. The samples were diluted to 1:128 and 1:256.

If a high positive result was observed, the samples were diluted to 1:512 for *R. conorii*, and to both 1:512 and 1:1024 for *E. canis*, *A. phagocytophilum* and *B. henselae* antigens. At this point, if the samples had not reached a dilution with a negative result, the samples were classified as a high positive for *R. conorii* (> 1:512) or as a high positive for *E. canis*, *A. phagocytophilum* or *B. henselae* antigens (> 1:1024).

### Blood DNA extraction and PCR for the detection of *Ehrlichia*, *Anaplasma* spp., *Hepatozoon* and *Babesia* spp. and filaroid DNA

Blood DNA extraction was performed as previously described [33, 35]. PCR was performed in samples from 76 of the 77 dogs included in the study: 60 with clinical leishmaniosis and 16 apparently healthy dogs.

### *Ehrlichia* and *Anaplasma* spp. DNA

Samples were screened in duplicates for the presence of *Ehrlichia*/*Anaplasma* DNA using primers which amplify a 123 bp of the *16S rRNA* gene of the genera *Anaplasm*a and *Ehrlichia* by a qPCR assay as previously described [36]. Positive samples from this reaction were further analysed in duplicates by conventional PCR using primers EHR16SD and EHR16SR which amplify a 345 bp fragment of the *16S rRNA* gene of species of the genera *Anaplasma* and *Ehrlichia* [37]. Positive and negative controls were included in both PCRs.

### *Hepatozoon* and *Babesia* spp. DNA

Detection of *Babesia* spp. and *Hepatozoon* spp. DNA was performed by a conventional PCR assay targeting a 400 bp fragment of the *18S rRNA* gene by using the following primers (3′-CCA GCA GCC GCG GTA ATT C-5′) and (3′-CTT TCG CAG TAG TTY GTC TTT AAC AAA TCT-5′) as described elsewhere [38]. All reactions were run in a StepOne Plus thermocycler (Applied Biosystems, Foster City, CA, USA). The samples were screened in duplicates, and positive and negative controls were included in each PCR run. Positive samples were tested by additional PCRs using primers specifically designed for the detection of a fragment of the *18S rRNA* gene of *Babesia* spp. (PIROA/PIROB) [39].

### Filarioid DNA

A high resolution melt (HRM) real-time PCR was performed as previously described [40] with some modifications. The qPCR was performed to amplify a partial sequence of the mitochondrial *12S* gene of filaroids of approximately 115 bp [41]. All reactions were run in duplicates in a StepOne Plus thermocycler (Applied Biosystems). Previously tested dog blood samples positive and negative for both canine filaroids were used as positive and negative controls, respectively.

### Sequencing PCR products

Samples that were positive by PCR were sequenced as described elsewhere [42]. Only sequences with identity between 97% and 100% and coverage above 99% were considered as positive for an organism.

### Statistical analysis

A descriptive study of the detection of antibodies, the number of co-infections detected in each dog, according to the results of the IFATs and PCRs performed, and the proportion of the antibody levels for each pathogen, was performed. The number of co-infections was calculated by the sum of the positive results for each test performed for each dog; therefore, the maximum number of co-infections was 7 (due to the fact that 4 IFATs and 3 PCRs were performed) and every dog had a result between 0 (no co-infections) and 7 (positive to all tests performed). Quantitative variables were assessed using the Mann-Whitney U-test and Spearman’s correlation. The Mann-Whitney U-test was used to compare the medians of quantitative variables of healthy and sick dogs. Spearman’s correlation was used to test for association between the number of co-infections detected and the clinical data of sick dogs that consisted of hematological and biochemical profile parameters, urinalysis with urinary protein creatinine ratio, and serum electrophoresis, the antibody levels in a quantitative ELISA for the detection of *L. infantum*-specific antibodies, the result for *Leishmania* qPCR and *L. infantum* IFN-γ concentration. Qualitative variables of healthy and sick dogs were assessed using Chi-quare, Fisher’s exact test, Kruskal-Wallis test and multivariable logistic regression. Fisher’s exact test was also used to compare the detection of antibodies for the different pathogens with the clinical signs observed in each sick dog. Kruskal-Wallis test was used to compare the number of co-infections detected with sex, age and season at time of diagnosis, and the proportion of the level of antibodies detected for each pathogen with clinical data of sick dogs. Multivariable logistic regression was used to correlate the detection of antibodies with the clinical data of sick dogs; every factor was included in the analysis and those that were less significant (*P-*value > 0.2) were excluded until all factors presented a *P-*value ≤ 0.2. The remaining factors were further studied using logistic regression. The Shapiro-Wilk test was performed to detect the normality of the distribution of the samples. A *P-*value < 0.05 was considered statistically significant. The statistical analysis was performed using the R program i386 version 3.3.1 (R Development Core Team) and the DeduceR program version 1.7–16 (Deducer: A data Analysis GUI for R) for Windows software.

## Results

### Signalment and clinical data

Both sexes were represented in the sick group with 37 males (61%) and 24 females (39%). Forty-two of 61 were intact, 30 males and 12 females. The median age at diagnosis was 5 years with a range from 5 months to 13 years. Forty-one dogs were purebred (67%) and 20 were classified as mixed-bred (33%).

The 61 dogs were classified in different stages of leishmaniosis after Solano-Gallego et al. [4]. Five (8%) were classified as stage I with mild disease; 43 (70%) were classified as stage II with moderate disease (31 classified as substage IIa and 12 classified in substage IIb); 10 (16%) were classified as stage III with severe disease; and 3 (5%) were classified as stage IV with very severe disease.

Both sexes were also represented in the healthy group with 5 males (31%) and 6 females (38%). Gender was not recorded in 5 dogs (31%). The median age at diagnosis was 7 years with a range from 15 months to 13 years. Seven dogs (44%) were purebred and 9 (56%) were classified as mixed-bred.

No statistical differences in age, sex or breed were found between sick and apparently healthy dogs.

### IFAT

#### Comparison of dogs with canine leishmaniosis and apparently healthy dogs

The results of IFAT for *R. conorii*, *E. canis*, *A. phagocytophilum* and *B. henselae* antigens in sick and healthy dogs studied as well as PCR results are shown in Table 1. The most frequent seropositive serology was for *R. conorii* (52/75; 69%), followed by *E. canis* (43/75; 57%), *A. phagocytophilum* (33/75; 44%) and *B. henselae* (28/75; 37%) antigens. Of the total 75 assessed by IFAT, 11 (15%) seroreacted with the 4 pathogen antigens, 16 (21%) with 3 pathogens, 24 (32%) with 2 of the pathogens screened and 16 (21%) which seroreacted with 1 pathogen. Consequently, 67 (89%) of the tested dogs seroreacted with at least 1 antigen by IFAT. Sera from 8 (11%) of the tested dogs did not react in any IFAT test performed. The pattern of results of IFAT in dogs with clinical leishmaniosis and healthy dogs is summarized in Table 2.

**Table 1:** Results of IFAT for *R. conorii*, *E. canis*, *A. phagocytophilum* and *B. henselae* antigens and PCR for *E. canis*, *Anaplasma* spp., *Hepatozoon* spp., *Babesia* spp. and filarioids in dogs with clinical leishmaniosis and healthy dogs. A comparison of the groups was made with Fisher’s exact test

Assays	Number (%, 95% CI) of seroreactive dogs			
IFAT	Dogs with clinical leishmaniosis (*n* = 61)	Healthy dogs (*n* = 14)	Total dogs (*n* = 75)	*P-*value
*R. conorii*	46 (75, 65–86)	6 (43, 17–69)	52 (69, 59–80)	0.025*
*E. canis*	34 (56, 43–68)	9 (64, 39–89)	43 (57, 46–69)	0.766
*A. phagocytophilum*	32 (53, 40–65)	1 (7, 0–21)	33 (44, 33–55)	0.002*
*B. henselae*	25 (40, 28–52)	3 (21, 0–43)	28 (37, 26–48)	0.147
PCR	Dogs with clinical leishmaniosis (*n* = 60)	Healthy dogs (*n* = 16)	Total dogs (*n* = 76)	*P-*value
*Ehrlichia*/*Anaplasma* spp*.*	8 (13, 5–22)	0 (0)	8 (11, 4–17)^a^	0.191
*H. canis* and *Babesia* spp*.*	1 (2, 0–5)	0 (0)	1 (1, 0–4)^b^	0.606
Filarioid DNA	0 (0)	0 (0)	0 (0)	–

*Abbreviations*: *CI* confidence interval, *IFAT* immunofluorescence antibody test, *PCR* polymerase chain reaction

^a^Two dogs remained with a positive result for *Anaplasma platys* after performing a conventional PCR and sequencing

^b^One dog was detected with *Hepatozoon canis* infection. No *Babesia* spp. or filarioid DNA were detected

^*^P-values statistically significant

**Table 2:** Pattern of IFAT results in dogs with clinical leishmaniosis and healthy dogs for one or more antigens (*R. conorii*, *E. canis*, *A. phagocytophilum* and *B. henselae*)

Antigens	Number (%, 95% CI) of seroreactive dogs			
	Dogs with clinical leishmaniosis (*n* = 57)	Healthy dogs (*n* = 10)	Total dogs (*n* = 67)	*P-*value^a^
*R. conorii* alone	6 (11, 3–19)	0 (0)	6 (9, 2–16)	0.58
*E. canis* alone	5 (9, 1–16)	2 (20, 0–45)	7 (10, 3–18)	0.279
*A. phagocytophilum* alone	2 (4, 0–8)	0 (0)	2 (3, 0–7)	0.548
*B. henselae* alone	1 (2, 0–5)	0 (0)	1 (1, 0–4)	0.673
*R. conorii* and *E. canis*	6 (11, 3–19)	4 (40, 10–70)	10 (15, 6–24)	0.035*
*R. conorii* and *A. phagocytophilum*	3 (5, 0–11)	0 (0)	3 (4, 0–9)	0.458
*R. conorii* and *B. henselae*	5 (9, 1–16)	1 (10, 0–29)	6 (9, 2–16)	0.9
*E. canis* and *A. phagocytophilum*	0 (0)	0 (0)	0 (0)	–
*E. canis* and *B. henselae*	0	2 (20, 0–45)	2 (3, 0–7)	0.02*
*A. phagocytophilum* and *B. henselae*	3 (5, 0–11)	0 (0)	3 (4, 0–9)	0.458
*R. conorii, E. canis* and *A. phagocytophilum*	10 (18, 8–27)	1 (10, 0–29)	11 (16, 8–25)	0.553
*R. conorii*, *E. canis* and *B. henselae*	2 (4, 0–8)	0 (0)	2 (3, 0–7)	0.548
*R. conorii*, *A. phagocytophilum* and *B. henselae*	3 (5, 0–11)	0 (0)	3 (4, 0–9)	0.458
*E. canis*, *A. phagocytophilum* and *B. henselae*	0 (0)	0 (0)	0 (0)	–
*R. conorii*, *E. canis*, *A. phagocytophilum* and *B. henselae*	11 (19, 9–30)	0 (0)	11 (16, 8–25)	0.195

*Abbreviations*: *CI* confidence interval

^a^Fisher’s exact test was performed

^*^P-values statistically significant

Fifty seven of the 61 (93%) dogs with clinical leishmaniosis had a positive result to at least one of the IFAT tests performed while 10 of the 14 (71%) dogs in the healthy group also had a positive result. A significant difference was found when comparing the two groups of dogs (Fisher’s exact test: *P* = 0.036, OR = 5.7, 95% CI = 1–35), thereby, dogs with clinical leishmaniosis were more likely to be positive to at least one of the IFAT compared to apparently healthy dogs. As shown in Table 1, the most frequent seropositivity in dogs with clinical leishmaniosis was for *R. conorii* while *E. canis* antibodies were the most frequent in the healthy group.

**Fig. 1:**
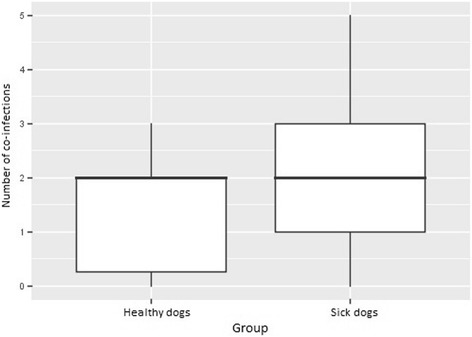
Comparison of the number of co-infections detected by IFAT and PCR between dogs with clinical leishmaniosis and apparently healthy dogs. A comparison of the means was performed with Mann-Whitney U-test (Mann-Whitney test: *U* = 243, *Z* = -2.6 *n*_*1*_ = 14, *n*_*2*_ = 61, *P* = 0.01)

Dogs with clinical leishmaniosis were more likely to have a positive result to more than one test (IFAT and PCR) (Mann-Whitney test:, *U* = 243, *Z* = -2.6, *n*_1_ = 14, *n*_2_ = 61, *P* = 0.01) (Fig. 1), to be seroreactive to *R. conorii* (Fisher’s exact test: *P* = 0.025, OR = 4.1, 95% CI = 1–17) and to *A. phagocytophilum* (Fisher’s exact test: *P* = 0.002, OR = 14.3, 95% CI = 2–626) antigens (Table 1) when compared with healthy dogs. No difference was found between seroreactivity to *E. canis* and *B. henselae* or being positive in the PCRs performed.

Of the 67 dogs that seroreacted to at least one pathogen, serial dilutions were performed, and the results are listed in Table 3. Healthy dogs were more likely to have a negative result or to have low antibody titers when compared with sick dogs (Table 3). Healthy dogs were likely to be negative for *R. conorii* (Fisher’s exact test: *P* = 0.025, OR = 4.1, 95% CI = 1–17) and *A. phagocytophilum* (Fisher’s exact test: *P* = 0.002, OR = 14.3, 95% CI = 2–626) antigens while a higher number of healthy dogs were positive at the antibody titer of 1:64 for *E. canis* when compared with sick dogs (Fisher’s exact test: *P* = 0.014, OR = 0.2, 95% CI = 0–0.9).

**Table 3:** IFAT antibody titers for *R. conorii*, *E. canis*, *A. phagocytophilum* and *B. henselae* antigens in dogs with clinical leishmaniosis and healthy dogs. Fisher’s exact test was performed

Antibody titers	Antigens	Number (%, 95% CI) of seroreactive dogs	
		Dogs with clinical leishmaniosis (*n* = 61)	Healthy dogs (*n* = 14)
< 64	R. conorii*	15 (25, 14–35)	8 (57, 31–83)
	*E. canis*	27 (44, 32–57)	5 (36, 11–61)
	A. phagocytophilum**	29 (48, 35–60)	13 (93, 79–100)
	*B. henselae*	36 (59, 47–71)	11 (79, 57–100)
64	*R. conorii*	12 (20, 10–30)	2 (14, 0–33)
	E. canis***	17 (28, 17–39)	9 (64, 39–89)
	*A. phagocytophilum*	17 (28, 17–39)	1 (7, 0–21)
	*B. henselae*	6 (10, 2–17)	1 (7, 0–21)
128	*R. conorii*	0 (0)	0 (0)
	*E. canis*	3 (5, 0–10)	0 (0)
	*A. phagocytophilum*	4 (7, 0–13)	0 (0)
	*B. henselae*	8 (13, 5–22)	1 (7, 0–21)
256	*R. conorii*	10 (16, 7–26)	0 (0)
	*E. canis*	3 (5, 0–10)	0 (0)
	*A. phagocytophilum*	3 (5, 0–10)	0 (0)
	*B. henselae*	10 (16, 7–26)	1 (7, 0–21)
512	*R. conorii*	15 (26, 15–37)	4 (29, 5–52)
	*E. canis*	2 (3, 0–8)	0 (0)
	*A. phagocytophilum*	4 (7, 0–13)	0 (0)
	*B. henselae*	0 (0)	0 (0)
> 512	*R. conorii*	9 (15, 6–24)	0 (0)
1024	*E. canis*	8 (13, 5–22)	0 (0)
	*A. phagocytophilum*	3 (5, 0–10)	0 (0)
	*B. henselae*	1 (2, 0–5)	0 (0)
> 1024	*E. canis*	1 (2, 0–5)	0 (0)
	*A. phagocytophilum*	1 (2, 0–5)	0 (0)
	*B. henselae*	0 (0)	0 (0)

*Abbreviation*: *CI* confidence interval

*P = 0.025, **P = 0.002, ***P = 0.014

Furthermore, a significant association was found in all dogs studied (healthy and sick dogs) between seroreactivity to *R. conorii* and seroreactivity to *E. canis* (Fisher’s exact test: *P* = 0.044, OR = 2.9, 95% CI = 1–9) or *A. phagocytophilum* (Fisher’s exact test: *P* = 0.012, OR = 4.2, 95% CI = 1–16), and seroreactivity to *A. phagocytophilum* and *E. canis* with high antibody titers (Chi-square test: *χ*^2^ = 26.36, *df* = 6, *P* < 0.001).

#### Comparison of dogs with canine leishmaniosis depending on co-infection status based on serological and molecular tests

All statistically-significant associations found between the pathogens tested and laboratory abnormalities and clinical signs in sick dogs are summarized in Table 4.

**Table 4:** Summary of signalment, clinical signs, laboratory abnormalities, and the results of diagnostic tests for leishmaniosis significantly associated with seroreactivity to different antigens tested by IFAT and with positive results tested by PCR in dogs with clinical leishmaniosis

Test performed	Pathogen	Laboratory abnormalities^a, b^	Clinical signs^c^
IFAT	*R. conorii* ^d^	Increase: ns Decrease: albumin, albumin/globulin ratio, lymphocyte concentration	ns
	*E. canis* ^e^	Increase: total protein, gamma globulins Decrease: albumin, albumin/globulin ratio, hematocrit, hemoglobin, RBC	ns
	*A. phagocytophilum* ^f^	Increase: total protein, beta globulins, gamma globulins Decrease: albumin, albumin/globulin ratio	No presence of lameness
	*B. henselae*	Increase: total protein, beta globulins, gamma globulins Decrease: albumin/globulin ratio, hematocrit, hemoglobin	Marked lymphadenomegaly
PCR	*Ehrlichia*/*Anaplasma*	Increase: ns Decrease: hematocrit, RBC, platelet concentration	ns
	*Hepatozoon*/*Babesia*	Increase: ns Decrease: ns	ns
Co-infections^g^		Increase: total protein, beta globulins, gamma globulins, UPC Decrease: albumin, albumin/globulin ratio, lymphocyte concentration, hematocrit, hemoglobin, MCH	ns

*Abbreviations*: *ns* non-significant, *UPC* urinary protein/creatinine ratio, *MCH* mean corpuscular hemoglobin

^a^All statistically significant associations are present in the result section of this manuscript

^b^No statistically significant association was found between the tested pathogens and other laboratorial abnormalities recorded (creatinine, urea, ALT, total cholesterol, urinary protein/creatinine ratio, leucocyte, monocyte, neutrophil, eosinophil and reticulocyte concentrations)

^c^No statistically significant association was found between the tested pathogens and the other clinical signs recorded (fever, weight loss, skin lesions, ocular lesions, muscular atrophy, splenomegaly, vomiting and diarrhea, joint pain, polyuria and polydipsia, epistaxis and neurological disorders)

^d^Statistically significant association was also found with older age (> 5 year-old) and a high positive antibody level by *L. infantum* quantitative ELISA

^e^Statistically significant association was also found with neutering

^f^Statistically significant association was also found with a high positive antibody level by *L. infantum* quantitative ELISA, being in stage III or IV of LeishVet clinical staging for *L. infantum* and being diagnosed in spring or winter

^g^Statistically significant association was also found with high parasite load of *L. infantum* and older age

**Fig. 2:**
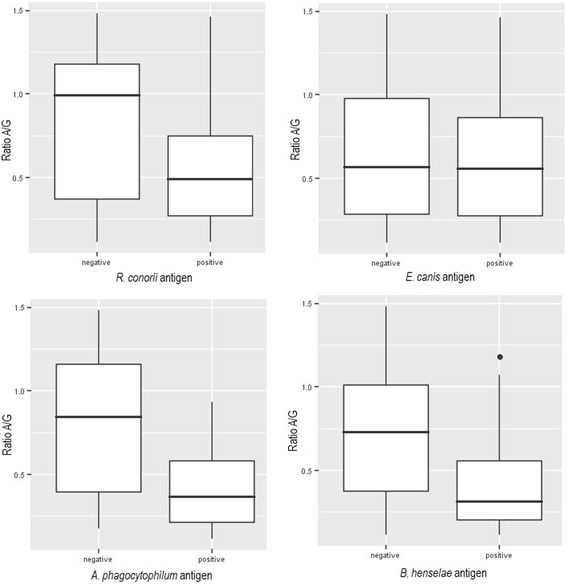
The relationship of the albumin/globulin ratio with the results (positive or negative) of the different IFAT performed at a dilution of 1:64. A comparison of the means was performed with logistic regression with the following results: *R. conorii* (*χ*^2^ = 5.27, *P* = 0.0217, OR = 0.2), *E. canis* (*χ*^2^ = 0.07, *P* = 0.7864, OR = 0.8), *A. phagocytophilum* (*χ*^2^ = 12.77, *P* = 0.0003, OR = 0) and *B. henselae* (*χ*^2^ = 7.98, *P* = 0.0047, OR = 0.1) antigens

**Fig. 3:**
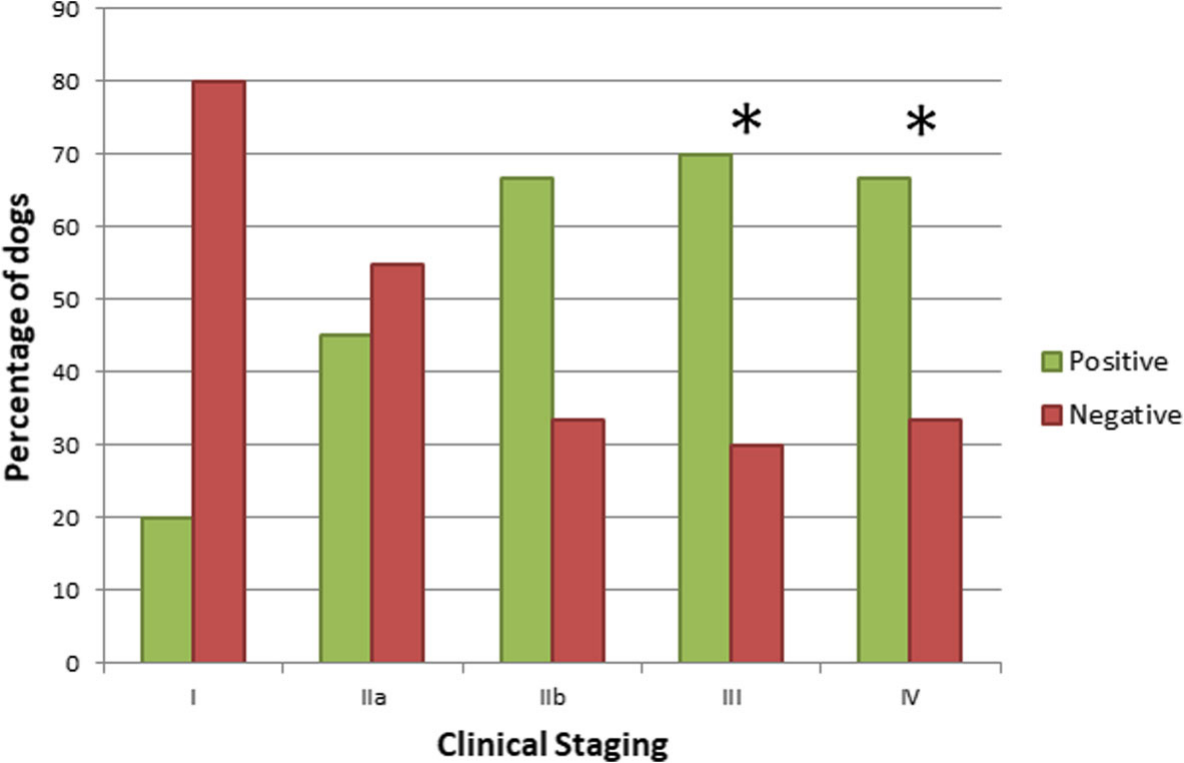
Results of IFAT for *A. phagocytophilum* antigen in dogs with clinical leishmaniosis based on the LeishVet clinical staging. Fisher’s exact tests were performed; asterisks indicate *P* = 0.042

When dogs with clinical leishmaniosis were compared with the same sick group depending on each pathogen specific seroreactivity (sick dogs seroreactive to one antigen vs sick dogs seronegative to the same pathogen), no statistical association was found between sex or the blood parasite load of *L. infantum* and any of the pathogens tested by IFAT. The presence of *R. conorii* antibodies was more frequent among sick dogs that were older than 5 years-old at time of diagnosis (Logistic regression: *χ*^2^ = 8.47, *df* = 1, *P* = 0.0036, OR = 1.03), sick dogs with a lower albumin/globulin ratio than the average of the sick group (Logistic regression: *χ*^2^ = 5.27, *df* = 1, *P* = 0.0217, OR = 0.2) (Fig. 2), sick dogs with a lower concentration of lymphocytes than the average of the group (Logistic regression: *χ*^2^ = 4.66, *df* = 1, *P* = 0.0309, OR = 0.9) and a high positive antibody level by the *L. infantum* quantitative ELISA (Chi-square test: *χ*^2^ = 13.04, *df* = 3, *P* = 0.005). The presence of *E. canis* antibodies was only associated with sick neutered dogs (Chi-square test: *χ*^2^ = 6.84, *df* = 1, *P* = 0.033) while the presence of *A. phagocytophilum* antibodies was more frequent in sick dogs with an increase of total protein (Logistic regression: *χ*^2^ = 4.64, *df* = 1, *P* = 0.0312, OR = 1.3), beta globulins (Logistic regression: *χ*^2^ = 4.28, *df* = 1, *P* = 0.0385; OR = 3.6) and gamma globulins (Logistic regression: *χ*^2^ = 5.37, *df* = 1, *P* = 0.0204, OR = 1.5) compared to the average of the tested sick group, a decrease of albumin (Logistic regression: *χ*^2^ = 9.82, *df* = 1, *P* = 0.0017, OR = 0.2), lower albumin/globulin ratio (Logistic regression: *χ*^2^ = 12.77, *df* = 1, *P* = 0.0003, OR = 0) (Fig. 2) compared to the average of sick group, a high positive antibody level by the *L. infantum* quantitative ELISA (Chi-square: *χ*^2^ = 13.04, *df* = 3, *P* = 0.003) and dogs classified in stage III or IV of the LeishVet clinical staging for *L. infantum* (Chi-square: *χ*^2^ = 9.38, *df* = 4, *P* = 0.042) (Fig. 3) and being diagnosed in spring or winter (Chi square: *χ*^2^ = 10.59, *df* = 3, *P* = 0.014). The presence of *B. henselae* antibodies in sick dogs was associated with an increase of total protein (Logistic regression: *χ*^2^ = 11.67, *df* = 1, *P* = 0.0006, OR = 1.8), beta globulins (Logistic regression: *χ*^2^ = 10.44, *df* = 1, *P* = 0.0012, OR = 2.3) and gamma globulins (Logistic regression: *χ*^2^ = 6.75, *df* = 1, *P* = 0.0094, OR = 1.5), a low albumin/globulin ratio (Logistic regression: *χ*^2^ = 7.98, *df* = 1, *P* = 0.0047, OR = 0.1), hematocrit (Logistic regression: *χ*^2^ = 7.1, *df* = 1, *P* = 0.0077, OR = 0.9) and hemoglobin (Logistic regression: *χ*^2^ = 6.72, *df* = 1, *P* = 0.0095, OR = 0.8).

When dogs with clinical leishmaniosis were compared with dogs from the same group that had different antibody titers (< 1:64, 1:64, 1:128, 1:256, 1:512; 1:1024 and > 1:1024 for *E. canis*, *A. phagocytophilum* and *B. henselae* and < 1:64, 1:64, 1:128, 1:256, 1:512 and > 1:512 for *R. conorii*), no significant association was found between sex, age, clinical signs and the clinical stage of leishmaniosis. A comparison between the average of different antibody titers was performed. The sick dogs that had a high positive (> 1:512) antibody titer for *R. conorii* antigen were positively associated with a decrease in albumin (Kruskal-Wallis H-test: *χ*^2^ = 12.98, *df* = 4, *P* = 0.0113), while a decrease of the albumin/globulin ratio was associated with an increase of antibody titers for *R. conorii* antigen (Kruskal-Wallis H-test: *χ*^2^ = 12.5, *df* = 4, *P* = 0.014). Furthermore, sick dogs that had antibody titers for *R. conorii* antigen at 1:256 and 1:512 dilutions were associated with production of IFN-γ after blood stimulation with *L. infantum* antigen (Chi-square test: *χ*^2^ = 10.74, *df* = 4, *P* = 0.028). These dogs with leishmaniosis were also associated with being diagnosed in the autumn (Chi-square test: *χ*^2^ = 11.44, *df* = 3, *P* = 0.011). No other pathogens were associated with the production of IFN-γ after blood stimulation with *L. infantum* antigen.

A significant association was found between a high *E. canis* antibody titer (> 1:512) and a decrease in albumin (Logistic regression: *χ*^*2*^ = 6.88, *df* = 1, *P* = 0.0087, OR = 0.2), albumin/globulin ratio (Logistic regression: *χ*^2^ = 7.24, *df* = 1, *P* = 0.0071, OR = 0), hematocrit (Logistic regression: *χ*^*2*^ = 7.97, *df* = 1, *P* = 0.0048, OR = 0.7), hemoglobin (Logistic regression: *χ*^*2*^ = 7.76, *df* = 1, *P* = 0.0053, OR = 0.6), RBC (Logistic regression: *χ*^2^ = 6.14, *df* = 1, *P* = 0.0132, OR = 0.1) and an increase in gamma globulins (Logistic regression: *χ*^2^ = 8.06, *df* = 1, *P* = 0.0045, OR = 2.4) and total protein (Logistic regression: *χ*^2^ = 9.81, *df* = 1, *P* = 0.0017, OR = 3). Furthermore, a significant association was found between high *A. phagocytophilum* antibody titers (> 1:512) and a decrease in albumin (Kruskal-Wallis H-test: *χ*^2^ = 21.68, *df* = 6, *P* = 0.0014) and the albumin/globulin ratio (Kruskal-Wallis H-test: *χ*^2^ = 21.65, *df* = 6, *P* = 0.0014).

When dogs with clinical leishmaniosis were compared with the same sick group according to the antibody titers, no statistical association was found between high *B. henselae* antibody titers and clinicopathological abnormalities.

When dogs with clinical leishmaniosis were compared with dogs from the same group that did not show the same clinical signs, no statistically-significant association was found between clinical signs and a positive result for *R. conorii* and *E. canis* tested by IFAT. Lymphadenomegaly was the only clinical sign significantly associated with *B. henselae* tested by IFAT (Fisher’s exact test: *P* = 0.044, OR = 4, 95% CI = 1–19). In addition, *B. henselae* seroreactivity was associated with marked lymphadenomegaly in sick dogs (Logistic regression: *χ*^2^ = 8.1, *df* = 1, *P* = 0.0044, OR = 2.3). Interestingly, *A. phagocytophilum* seroreactivity was associated with a lower probability of suffering from lameness (Fisher’s exact test: *P* = 0.021, OR = 0, 95% CI = 0–0.9) when compared with sick dogs that did not seroreact with *A. phagocytophilum* antigen.

When dogs with clinical leishmaniosis were compared with dogs from the same group divided according to the number of co-infections (as detected by both IFAT and PCR), no significant association was found between sex or the clinical stage of leishmaniosis. A significant association between the number of co-infections and the blood parasite load of *L. infantum* (*r*_(66)_ = 0.3, *P* = 0.0252), age at time of diagnosis (*r*_(67)_ = 0.2, *P* = 0.0496), total protein (*r*_(68)_ = 0.4, *P* = 0.0005), urinary protein/creatinine ratio (UPC) (*r*_(42)_ = 0.3, *P* = 0.0256), albumin (*r*_(66)_ = -0.4, *P* = 0.0008), albumin/globulin ratio (*r*_(62)_ = -0.5, *P* = 0.0001), beta globulins (*r*_(56)_ = 0.4, *P* = 0.0025), gamma globulins (*r*_(56)_ = 0.4, *P* = 0.002), hematocrit (*r*_(56)_ = -0.3, *P* = 0.0446), hemoglobin (*r*_(57)_ = -0.3, *P* = 0.045), mean corpuscular haemoglobin (MCH) (*r*_(48)_ = -0.4, *P* = 0.0074) and lymphocyte concentration (*r*_(54)_ = -0.3, *P* = 0.0493) was found by Spearman’s correlation in sick dogs.

### PCR

#### Detection of *Ehrlichia* and *Anaplasma* spp. DNA

Of the 60 dogs with clinical leishmaniosis assessed, 8 (10.5%) were positive for *Ehrlichia* and *Anaplasma* by real-time PCR. However, positive amplicons for *E. canis* and *Anaplasma* qPCR did not produce a conclusive sequence. Of those 8 dogs, only 2 (2/8; 25%) maintained a positive result after performing a conventional PCR. Sequencing showed that both dogs were detected with *A. platys* (Table 5). All apparently clinically healthy dogs resulted negative.

**Table 5:** Dogs positive by the different PCRs performed and the corresponding IFAT results for different antigens studied

Dog ID	qPCR *L. infantum* (parasite/ml); ELISA dilution (EU)	IFAT				qPCR *Ehrlichia*/ *Anaplasma*	Conventional PCR *Ehrlichia*/ *Anaplasma*	PCR *Hepatozoon*/ *Babesia*	Clinical signs and laboratory abnormalities
		*Rc*	*Ec*	*Ap*	*Bh*				
HCV-7	Negative (0); Positive (195)	512	1024	1024	256	Positive	Negative	Negative	Moderate lymphadenomegaly, skin lesions. Thrombocytopenia, hypercholesterolemia, hyperproteinemia, hyperbetaglobulinemia
HCV-9	Positive (51); Positive (39892)	< 64	< 64	< 64	256	Positive	Negative	Negative	Fever, weight loss, moderate lymphadenomegaly, ocular lesions. Pancytopenia, hypoalbuminemia, hypergammaglobulinemia
HCV-11	Positive (4); Positive (12568)	> 512	< 64	64	128	Positive	Negative	Negative	Skin lesions. Hypoalbuminemia, hypergammaglobulinemia, proteinuria
HCV-28	Positive (807); Positive (30827)	512	1024	256	< 64	Positive	Negative	Negative	Weight loss, moderate lymphadenomegaly, skin and ocular lesions. Regenerative anemia, hypoalbuminemia, hypergammaglobulinemia, proteinuria
HCV-31	Positive (8); Positive (37360)	64	64	128	64	Positive	Negative	Negative	Fever, mild lymphadenomegaly, epistaxis. Anemia, lymphocytosis, thrombocytopenia, hypoalbuminemia, hypergammaglobulinemia
MO-1	Positive (33); Positive (47)	512	128	64	< 64	Positive	Positive^a^	Negative	Skin lesions. Hypercholesterolemia
MO-4	Negative (0); Positive (188)	256	64	1024	< 64	Negative	–	Positive^a^	Skin lesions. Mild hyperbetaglobulinemia
MED-5	Negative (0); Positive (7170)	64	< 64	< 64	256	Positive	Positive^a^	Negative	Weight loss, moderate lymphadenomegaly, skin and ocular lesions, splenomegaly. Anemia, hypoalbuminemia
MED-7	Negative (0); Positive (82)	< 64	< 64	1024	< 64	Positive	Negative	Negative	Moderate lymphadenomegaly, skin lesions. Hypoalbuminemia

*Abbreviations*: *Ap Anaplasma phagocytophilum*, *Bh Bartonella henselae*, *Ec Ehrlichia canis*, *IFAT* immunofluorescence antibody test, *PCR* polymerase chain reaction, *Rc Rickettsia conorii*, *qPCR* real-time PCR

^a^Three pathogens were sequenced with the following results: two *A. platys* and one *H. canis*

When comparing the results of the PCR between dogs with clinical leishmaniosis and healthy dogs with Fisher’s exact test, no difference was found between the groups.

When dogs with clinical leishmaniosis that were PCR positive were compared to PCR negative sick dogs, a statistically significant association was found between a PCR positive result and a decreased hematocrit (Logistic regression: *χ*^2^ = 4.8, *df* = 1, *P* = 0.0281, OR = 0.9), RBC (Logistic regression: *χ*^2^ = 3.9, *df* = 1, *P* = 0.048, OR = 0.4) and platelet concentration (Logistic regression: *χ*^2^ = 3.98, *df* = 1, *P* = 0.0461, OR = 0.9).

No significant association was found between the origin of the dogs (Barcelona or Tarragona) and a positive result by PCR, although the two dogs that had a positive result in the conventional PCR were from Tarragona.

#### Detection of *Hepatozoon* spp., *Babesia* spp. and filarioid DNA

Of the 77 dogs assessed, only 1 (1.3%) had a positive result by PCR for *Hepatozoon* spp. This dog was diagnosed with clinical leishmaniosis. After sequencing, the pathogen found was *H. canis* (Table 5). *Babesia* and filarioid DNA were not detected in any of samples studied.

No statistically significant association was found between the positive *H. canis* PCR result and any of the clinical characteristics of the sick dogs assessed.

## Discussion

Previous studies have suggested that CanL could be affected by other vector-borne pathogens. De Tommasi et al. [26] found that infection with two or more vector-borne pathogens could complicate the clinical presentation and severity of hematological abnormalities in dogs with vector-borne disease. Mekuzas et al. [30] examined naturally exposed dogs with *L. infantum* and *E. canis* co-infection and proposed that the increase in clinical signs in co-infection is associated with a synergistic pathological effect between both pathogens. Furthermore, it was suggested that *E. canis* infection could contribute to the establishment of CanL [30]. In addition, Baneth et al. [27] examined three dogs with *E. canis* and *H. canis* co-infection in the same host cell and suggested that infection with one pathogen could permit or enhance invasion of another. Conversely, Tabar et al. [43] examined dogs with leishmaniosis and filariosis to detect filarial spp., *Wolbachia* spp. and *Leishmania* co-infection, and although an increase of disease severity and clinical signs was observed with *Leishmania*-filarial co-infection, it was also suggested that *Wolbachia* could have a protective role against *Leishmania* infection.

The present study demonstrated the presence of co-infections with vector-borne pathogens in dogs with clinical leishmaniosis living in the Mediterranean basin. To the best of our knowledge, a statistically significant relationship between sick dogs and a higher proportion of co-infections with the detection of *R. conorii* or *A. phagocytophilum* antibodies, when compared with healthy dogs, was found for the first time. In agreement with these results, a recent study documented that co-infection with several tick-borne pathogens caused clinical progressions of leishmaniosis in foxhounds in the USA [44]. In disagreement with previous reports [26, 30, 45–47], no association was found between seroreactivity to *E. canis* antigen and sick dogs with leishmaniosis. Interestingly, a positive trend was noted in our study in the association between seroreactivity to *B. henselae* antigen and sick dogs when compared with healthy dogs, although this was not statistically significant. A previous study found a significant percentage of seroreactivity to *Bartonella* antigens in sick dogs with clinical signs compatible with vector-borne diseases when compared to clinically healthy dogs in the USA where a large number of dogs were evaluated [48].

This study showed that more marked clinicopathological abnormalities such as decrease in albumin or RBC numbers or increase in globulins were noted in dogs with clinical leishmaniosis with a higher number of co-infections compared to dogs with CanL and a lower number of co-infections. This is in agreement with previous studies [49–51]. Those studies demonstrated more marked thrombocytopenia, an evident reduction of platelet aggregation response, a significant increase in activated partial thromboplastin time (APTT) and a reduction of the albumin/globulin ratio in dogs with clinical leishmaniosis co-infected with *E. canis* [49–51]. Here, in the present study, we report for the first time that certain clinicopathological abnormalities are more marked in dogs with co-infections based on positive serology for *R. conorii*, *A. phagocytophilum*, *E. canis* and *B. henselae*. It is important to highlight that based on the present findings, moderate to marked hypoalbuminemia or hyperglobulinemia in dogs with clinical leishmaniosis should arouse the suspicion of co-infections with other vector-borne pathogens. It has been shown that infection with tick-borne pathogens such as *R. conorii*, *B. henselae*, *A. platys*, *A. phagocytophilum* and *E. canis* may induce a decrease in serum concentration of negative acute phase proteins and an increase of positive acute phase proteins [19, 52–55]. Albumin is a negative acute phase protein whose level tends to decrease in inflammation or infection [56, 57].

Furthermore, it is noteworthy to mention that pathogen DNA was only detected in dogs with clinical leishmaniosis although no significant difference in detection was found when comparing with healthy dogs. *Anaplasma platys* and *H. canis* were confirmed as infecting dogs with clinical leishmaniosis by PCR. Interestingly, a significant association was found between dogs positive for *E. canis* and *Anaplasma* spp. by PCR and a low hematocrit, RBC and platelet concentration, which are typical clinicopathological findings in canine ehrlichiosis or anaplasmosis that could be worsened due to the co-infection [16, 54, 58, 59].

In this study, clinical signs common in leishmaniosis such as skin lesions, progressive weight loss, generalized lymphadenomegaly or splenomegaly were also evaluated. Lymphadenomegaly was the only clinical sign statistically associated with being seroreactive to *B. henselae* antigen. Furthermore, seroreactivity to *B. henselae* antigen was also positively associated with the degree of severity of lymphadenomegaly classified as mild, moderate or marked. Lymphadenomegaly is common in both diseases, leishmaniosis and bartonellosis [2, 4, 21]. Interestingly, an association was found between antibodies against *A. phagocytophilum* and more advanced clinical stages of leishmaniosis (LeishVet stage III and IV) in agreement with a recent study [44]. Further studies are needed to understand the relationship between co-infections and clinical leishmaniosis in dogs.

Previous studies have evaluated the serological and molecular evidence of exposure to vector-borne pathogens in dogs in Catalonia (Spain) [7, 8, 10, 12]. When comparing our results to those of these studies, we found a high increase of seropositivity rates to other pathogens when studying dogs with clinical leishmaniosis. For example, the seroprevalence found for *E canis* in our study was 56% in dogs with clinical leishmaniosis, while those other studies found seroprevalences of 16.7% [8] and 5% [12] for *E. canis* in healthy dogs. Interestingly, the seroprevalence found for *Bartonella* spp. was rather similar to that found in other studies carried out in Catalonia and the Island of Mallorca. Roura et al. [7] found a seroprevalence of 28% for *B. vinsonii berkhoffii* while another study found a seroprevalence of 16.8% for *B. henselae* and 1.1% for *B. vinsonii berkhoffii* [8].

Combining the serological and molecular results of the present study with the findings from previous literature, it is noteworthy to remark that co-infections patterns are different in several geographical regions where the dogs with leishmaniosis live and there is variability in their life style, exposure to ticks and fleas, the species of ectoparasites present in the area, and also on the preventative measures applied against ticks and fleas. For example, in the present study, *A. platys* and *H. canis* were only confirmed by PCR in dogs from the Tarragona area. In the Mediterranean basin, where mosquitoes and *R. sanguineus* (*s.l.*) ticks are common, it would be expected that the pathogens related to this tick species would be also prevalent [15, 24, 60]. However, comparing the present study with other recent studies from Croatia [61], Greece [62, 63], Corsica [64], Cyprus [65], Tunisia [66] and Israel [67], it is evident that *E. canis*, *Hepatozoon* spp. *Babesia* spp. and *Dirofilaria* spp. are circulating abundantly in those countries while, the results suggest that they are less common in Catalonia.

PCR is a technique that detects pathogen DNA and can, therefore, confirm infection although a negative result does not completely exclude it. Serological techniques, such as ELISA and IFAT, on the other hand, detect antibodies formed due to current infection or past exposure to the pathogen studied. Quantitative serology may be used to detect seroconversion, but seropositivity may also result from cross-reaction with antibodies formed against other organisms with similar antigens. PCR also allows identification of the pathogen. Due to the aforementioned characteristics, it is recommended to use both techniques for diagnosis of some infectious diseases [10, 68, 69]. In the present study, the results for the different PCR performed had some important limitations in the detection of positive samples, possibly due to the low pathogen load in the blood. It is important to note that with the particular pathogens studied, serial evaluations of blood parasitaemia or bacteraemia by PCR are recommended to enhance the likelihood of PCR detection [70]. In the present study, no repeated testing of the same dogs was performed and serology was not used to detect seroconversion, although seroconversion could have been helpful in the detection of a higher number of acute infections [19, 70]. Furthermore, in the present study, no PCR was performed to detect *Rickettsia* spp. such as *R. conorii* due to the low rickettsiaemia usually found in dogs [10, 19, 71]. PCR to detect *Bartonella* was also not performed. These bacteria are often cultured with an enrichment medium for insect cell culture growth (BAPGM) prior to PCR testing to increase the likelihood of detecting this species [72].

The different cross-reactions that could have occurred in this study should also be considered. It has been reported that positive reaction found in serological tests for *R. conorii* in dogs might be due to infection with other spotted fever group (SFG) *Rickettsia* spp. such as *R. massiliae*, *R. slovaca* or *R. aeschlimannii*, which are common in ticks in the Mediterranean basin countries [19, 73, 74]. Furthermore, serological cross-reactivity between *A. phagocytophilum* and *A. platys* is common, due to their antigenic similarity [12, 75, 76]. In Europe, *A. phagocytophilum* is usually transmitted by *I. ricinus* ticks while *A. platys* is suspected to be transmitted by *R. sanguineus* (*s.l.*) [15–17]. Taking into account that the main tick that inhabits the Barcelona area is *R. sanguineus* (*s.l.*) [77], it can be suggested that the positive serological reactivity was probably aimed at *A. platys* and not *A. phagocytophilum*. Similarly, *E. canis* can also have some degree of cross-reactivity with *Anaplasma* spp. [78, 79]. In the present study, 22 dogs seroreacted to both, *E. canis* and *A. phagocytophilum*, without positive PCR and sequencing. It could be suggested that these dogs were exposed only to one of the two vector-borne pathogens detected and they could have been infected by *A. platys*, the only Anaplasmataceae species detected by PCR. In addition, other species of *Bartonella* apart from *B. henseale* such as *Bartonella vinsonii berkhoffii* are associated with clinical illness in dogs. Therefore, the present study might have detected *Bartonella* seroreactivity related to infection with these other *Bartonella* species [80].

Another finding of the present study was the detection of a higher number of pathogens by IFAT and PCR in older dogs compared to young dogs. It is reasonable that older dogs would have more time and opportunity to be exposed to the different pathogens studied, although young dogs could be more susceptible to infections due to the immaturity of the immune system [81–84]. In agreement, Amusategui et al. [9] found that *R. conorii* infection was significantly associated with older age. However, a recent study [85] found that young animals are more susceptible to co-infection of *Leishmania* and *Babesia* spp. and Miró et al. [12] found that dogs under one year of age showed higher seropositivity rates for *E. canis* and *Borrelia burgdorferi* compared to dogs older than one year. Further studies are needed to understand the relationships between age and different vector-borne diseases, taking into account other factors such as lifestyle and location.

When studying vector-borne pathogens, it is also expected to find a relationship between the time of infection detection and the season when the vector is more active. In this study, only the results of IFAT for *A. phagocytophilum* antigen showed an association between seropositivity and season, in this case spring or winter. The vector for *A. phagocytophilum* present in Spain is the *I. ricinus* tick [15–17], which has the highest activity between April and June, a decrease of activity thereafter and a slight increase in the autumn-winter months [86]. When evaluating our results, it could be suggested that dogs with a positive IFAT for *A. phagocytophilum* were infested with these ticks and a subsequent infection occurred. However, *I. ricinus* is usually not found in the Mediterranean area [15, 76, 77, 86] and it parasites dogs only in rare cases since its natural hosts are wild animals such as rodents, wild boars and wild ruminants [15, 77]. Consequently, antibodies reactive with *A. phagocytophilum* are likely to have been formed against *A. platys*, for which the tick *R. sanguineus* (*s.l)* is suspected as its main vector. Different studies [76, 87, 88] have evaluated the seasonal dynamics of this tick in the Mediterranean basin and, although it has been stated that the highest activity of *R. sanguineus* (*s.l.*) is in summer, this tick can infest dogs during all seasons [76, 87]. Furthermore, *A. platys* is known to cause subclinical infections [16, 55, 89] and in fact the detection of this infection might not be associated with a certain season. On the other hand, no association was found between other vector-borne pathogens and seasonality. This could be due to the high probability of subclinical or chronic infection with *E. canis* [16] with the consequent delay in detection of infection as well as with leishmaniosis [2, 4, 6].

## Conclusions

This study demonstrates that dogs with clinical leishmaniosis from the Barcelona and Tarragona areas have a higher rate of co-infections with other vector-borne pathogens when compared with healthy controls. Furthermore, individual seroreactivity to *R. conorii*, *E. canis*, *A. phagocytophilum* and *B. henselae* antigens was associated with more pronounced clinicopathological abnormalities when compared with sick dogs that were seronegative to the same individual antigen. Interestingly, only seroreactivity of leishmaniotic dogs to *A. phagocytophilum* was associated with increased disease severity of clinical leishmaniosis.

## Abbreviations

ALT: Alanine aminotransferase
APTT: Activated partial thromboplastin time
BAPGM: *Bartonella*/alpha-Proteobacteria growth medium
CanL: Canine leishmaniosis
CBC: Complete blood cell count
CI: Confidence interval
ConA: Concavalin A
Ct: Cycle threshold
EDTA: Ethylenediaminetetraacetic acid
ELISA: Enzyme-linked immunosorbent assay
EU: ELISA unit
H2O: water
H2SO4: sulfuric acid
HRM: High resolution melting
IFAT: Immunofluorescence antibody test
IgG: Immunoglobulin G
LSA: *L. infantum* soluble antigen
MCH: Mean corpuscular hemoglobin
NTC: Non-template control
OD: Optical density
OR: Odds ratio
PBS: Phosphate-buffered saline
PCR: Polymerase chain reaction
qPCR: real-time PCR
RBC: Red blood cells
SD: Standard deviation
SFG: Spotted fever group
SPF: Specific pathogen free
UPC: Urinary protein/creatinine ratio
UV: Ultraviolet
